# A Secure Cluster-Based Multipath Routing Protocol for WMSNs

**DOI:** 10.3390/s110404401

**Published:** 2011-04-15

**Authors:** Islam T. Almalkawi, Manel Guerrero Zapata, Jamal N. Al-Karaki

**Affiliations:** 1 Computer Architecture Department, Technical University of Catalunya, Barcelona 08029, Spain; E-Mail: guerrero@ac.upc.edu; 2 Computer Engineering Department, The Hashemite University, Zarqa 13111, Jordan; E-Mail: jkaraki@hu.edu.jo

**Keywords:** wireless multimedia sensor networks, WMSN, WSN, routing protocol, multimedia delivery, multipath, cluster-based routing, security

## Abstract

The new characteristics of Wireless Multimedia Sensor Network (WMSN) and its design issues brought by handling different traffic classes of multimedia content (video streams, audio, and still images) as well as scalar data over the network, make the proposed routing protocols for typical WSNs not directly applicable for WMSNs. Handling real-time multimedia data requires both energy efficiency and QoS assurance in order to ensure efficient utility of different capabilities of sensor resources and correct delivery of collected information. In this paper, we propose a Secure Cluster-based Multipath Routing protocol for WMSNs, SCMR, to satisfy the requirements of delivering different data types and support high data rate multimedia traffic. SCMR exploits the hierarchical structure of powerful cluster heads and the optimized multiple paths to support timeliness and reliable high data rate multimedia communication with minimum energy dissipation. Also, we present a light-weight distributed security mechanism of key management in order to secure the communication between sensor nodes and protect the network against different types of attacks. Performance evaluation from simulation results demonstrates a significant performance improvement comparing with existing protocols (which do not even provide any kind of security feature) in terms of average end-to-end delay, network throughput, packet delivery ratio, and energy consumption.

## 1. Introduction

In this section, we introduce briefly two major issues of designing WMSNs, which are routing and security in WMSNs, examine their main challenges, and present how our novel proposed solutions address them.

### 1.1. Routing in WMSNs

Routing in Wireless Multimedia Sensor Networks (WMSNs) is very challenging due to several characteristics and constraints that distinguish them from contemporary communication and traditional wireless sensor networks [1,2]. *For example*, classical IP-based routing protocols cannot be applied to WMSNs to build a global addressing scheme for a network of large number of sensor nodes. *Also*, sensor nodes are tightly constrained in terms of available energy, processing power, available bandwidth and storage capacity and thus require careful resource management. *Moreover*, the multimedia nature of the collected information in WMSN (video streaming, audio, and still images) adds more constraints on the design of the routing protocols in order to meet the application-specific QoS requirements and network conditions, and in consequence makes the proposed routing protocols for WSNs not directly applicable for WMSNs. *In addition*, the use of densely deployed sensor nodes provides significant redundancy in the collected sensor data, e.g., overlapping of FoVs (Field of Views) of two camera sensors located in the same cluster. In that case, the two cameras may collectively fuse their information to obtain a lower bandwidth aggregated result, which is then routed (and possibly fused with other sensor node readings along the way or within the cluster) to a cluster head or the sink. Therefore, such redundancy needs to be exploited by the routing protocols to improve energy and bandwidth utilization and for more accurate and robust observation results through effective data fusion and aggregation.

Handling multimedia content in WMSNs produces many traffic classes and they can be categorized into three main classes or services depending on their QoS requirements: (1) Event-driven service which is delay intolerant and error intolerant but it requires less bandwidth, so a path with a little traffic and high signal to noise ratio is attractive for this kind of service. (2) Data query service is error intolerant but query-specific delay tolerant applications, so a path with significant congestion and a high signal to noise ratio may be used for this service. (3) Stream query service which is delay intolerant but query-specific error tolerant application (in a sense packet losses can be tolerated to a certain extent), so a path with less traffic and relatively lower signal to noise ratio is better for this type of service.

Due to the above mentioned challenges and differences, new routing protocols have been proposed for WMSNs [1] that try to handle these new characteristics of WMSN and its design challenges by either modifying the previous work done in WSNs (e.g., using multiple performance metrics to meet the additional QoS requirements), or proposing new solutions based on new methodologies (e.g., using multi radio or MIMO systems, switching between multiple channels, selecting multi routing paths, or mixing among these methods).

For WMSNs, cluster-based network architecture deployed with heterogeneous sensor nodes has more advantages than a flat network of homogeneous nodes. A single-tier flat architecture can cause the sink to overload with the increase in sensors density, which can affect the performance of the network and cause latency in communication and tracking events. Also, in the homogeneous flat network, all the nodes should have the same hardware capabilities and functionalities for multimedia processing and transmission, and this leads to increase the energy consumption and the cost of the deployed network. Therefore in this paper, cluster-based with multi-path routing protocol has been pursued in our proposal to allow the network to cover a large area of interest and cope with additional load without degrading the quality of service. Our proposed routing protocol aims to cluster the nodes, so that cluster heads can do some aggregation and reduction of data in order to save energy consumption and bandwidth usage, and to find the maximum number of paths suitable for the different requirements of handling different traffic classes. More specifically, the proposed routing protocol aims to satisfy the following design goals: (1) supporting different traffic classes of different delay and bandwidth requirements by choosing the suitable path for each data type, (2) maintaining minimum end-to-end delay suitable for real-time and non-real-time data packets to meet their playout deadlines, (3) achieving high throughput and packet delivery ratio by selecting the paths with better link quality and avoiding collisions and interferences, (4) saving energy at sensor nodes by moving the multimedia processing complexity as well as the aggregation process to the cluster heads’ side, along with preventing path loops and path cycles in establishing the routes, (5) providing load balancing and reliable data delivery by using multi-path routing protocol and two-level QoS-aware scheduling, and (6) supporting a secure communication among the nodes by implementing a light-weight distributed security scheme for key management as explained in the following subsection.

### 1.2. Security in WMSNs

Many applications of WMSN have their additional and special requirements in terms of privacy and security, such as military applications, medical care applications, and other video surveillance systems. In addition to the fact that WMSNs are vulnerable to attacks more easily than the wired networks because of their nature as a broadcast medium. In a broadcast medium, adversaries can easily eavesdrop on, intercept, inject, and alter transmitted data [3]. In addition, adversaries are not restricted in using sensor network hardware. They can interact with the network from a distance by using expensive radio transceivers and powerful workstations. In general, there are many types of security attacks on sensor networks such as: jamming, tampering, altered routing information, sinkhole, Sybil, wormholes, acknowledgment spoofing, and hello-flood attacks [4].

Attacks can be classified into outsider attacks and insider attacks [4]. Outsider attacks are where the attacker has no special access to the sensor network, and the insider attacks are where an authorized participant in the sensor network has gone badly. Insider attacks can be either compromised sensor nodes running malicious code or external devices use stolen key material and data from legitimate nodes to attack the network.

The resource scarcity, *ad hoc* deployment, and immense scale of WMSNs make secure communication a particularly challenging problem. Since the primary consideration for sensor networks is energy efficiency, security schemes must balance their security features against the communication and computational overhead required to implement them. Therefore, our focus for designing the security scheme is to implement a lightweight security protocol in terms of energy efficiency and processing complexity in order to secure the communication among the nodes so that every eligible receiver should receive all messages intended for it and be able to verify the integrity of every message as well as the identity of the sender. In this paper, we propose a lightweight distributed key management protocol designed to ensure authentication, integrity and confidentiality, without needing of a central key distribution. Our security scheme is scalable because every node needs to generate a small number of security keys even with deploying a large-scale network, it protects against the majority of outsider attacks, and it resists against insider attacks since the stolen keys will affect only the local area (cannot be used in other clusters). Advanced insider attacks can be eliminated by using simple intrusion detection [5], which is outside the scope of this paper.

The rest of the paper is organized as the following: Section 2 introduces an overview of existing related works. Section 3 provides network architecture and system model and assumptions. Section 4 describes the cluster-based multipath routing protocol in details. Section 5 presents the security scheme for key management. Section 6 presents the performance evaluation. Finally, Section 7 concludes the paper with summary and directions for future works.

## 2. Related Work

### 2.1. Routing in WMSNs

In this subsection, we briefly survey the major and recent related works in multipath and cluster-based routing protocols for WMSNs. In [6], an extensive survey has been conducted on the routing techniques with QoS assurance, and also a classification for the methodologies used for the proposed routing protocols can be found in [1].

An energy and QoS-aware hierarchical routing protocol, PEMuR [7], is proposed to handle multimedia data over WMSNs. PEMuR uses video packet scheduling algorithm based on an analytical distortion prediction model to reduce the video transmission rate (dropping less significant packets) while keeping minimum accepted distortion. The adopted routing protocol enables the selection of the energy efficient routing paths, manages the network load according to the energy residues of the nodes and prevents useless data transmissions through the proposed use of an energy threshold. PEMuR assumes that the network is deployed with homogeneous nodes where the number of cluster heads is predefined and they are elected by a powerful base station that can transmit to all network nodes.

A multipath routing protocol for WMSNs is presented in [8] called MPDT. This Multipath Data Transfer protocol provides simultaneous multiple paths for communication between sources and destination. The routing discovery process starts from the sources toward the sink using discovery messages. MPDT aims to prolong the lifetime of the network by providing load balancing and to be immune to some specific attacks by splitting the data into parts and encoding them using Reed Solomon (RS) algorithm.

Another multipath and QoS routing protocol is proposed for WMSNs in [9] called REAR. In this protocol metadata is used instead of the real data to establish multi-path routing in order to reduce the energy consumption and delay. Route establishment starts from the sources when they have data to be sent by sending control messages to their neighbors containing the metadata. Intermediate nodes that are interested in those metadata forward the control messages toward the sink. Found routes are then optimized using modified version of Dijkstra algorithm to meet the QoS requirements for the real data.

Multimedia-aware Multipath Multi-Speed (Multimedia-aware MMSPEED) routing protocol is proposed in [10]. Multimedia-aware MMSPEED is an extension over MMSPEED routing protocol to take into account the embedded information in the received packets in which near optimum path is reserved for I-packets and marginal paths are used for P-frames. MMSPEED protocol [11], which is also an extension for SPEED protocol [12] that was designed for WSN, can differentiate between flows with different delay and reliability requirements and has significant potential in video transmission applications. However, experimental results in [10] show that MMSPEED is not compatible with some special features of Multimedia traffic such as high video frame rate and packets information dependency.

A non-interfering disjoint multipath routing for WMSN is proposed in [13]. It addressed the problem of interference between multiple paths using one channel in WMSN and suggested an incremental on-demand approach in which only one path is built for a given source and additional paths are built when required in case of path congestion or lack of bandwidth. In order to solve the problem of interference between close paths, the proposed solution forces the multipath routing to build paths that are not interfering with each other from the beginning by putting all the interfering nodes of a given path in a passive state. Passive nodes do not further participate in building any other path in future and consequently will not interfere with previously built paths. The process starts at the sink when it floods the network with requests until they reach the source nodes. The source node starts immediately sending data on the selected paths and all the intermediate nodes between the source and the sink will inform their neighbors to switch to the passive state. The proposed work argues that putting some nodes in a passive or sleep mode increases the overall throughput and reduces the consumed amount of energy in the network. Results obtained through simulation show that the proposed protocol achieves better throughput with less energy consumption by using fewer non-interfering paths when compared to multipath schemes without interference awareness.

Modifications on Directed Diffusion [14], the routing protocol for WSN, are done in [15], called DHCT, to support multipath routing for WMSN based on link quality and latency metrics. One of the modifications includes using Costp, which is a product of expected transmission count (ETX) and delay, as a performance metric instead of pure delay that was used in Direct Diffusion. Since close paths interfere with each other and consequently have poor SNRs which are indirectly used to estimate ETX values, they have less probability to be selected by using Costp metric and this also will lead to increase throughput. The other modification is to reinforcing multiple links at the sink to obtain disjoint path from the source, and to match multipath routing. However, this routing protocol does not consider the bandwidth as QoS metric for routing decision or prioritizes the incoming packets to schedule them but it does consider the playout deadline in a sense that the data arrives after the deadline will be discarded. Results show that the presented protocol for multipath video streaming over WSNs obtains higher throughput than its single path counterpart (EDGE [16]) through the use of multiple disjoint paths.

A design of QoS aware routing protocol is presented in [17] to support high data rate for WMSNs by ensuring bandwidth and end-to-end delay requirements of real time data and maximized throughput of non-real time data. The routing protocol uses multiple paths, multiple channels, and QoS packet scheduling technique based on the dynamic bandwidth adjustment and path-length-based proportional delay differentiation (PPDD) techniques to meet the bandwidth and delay requirements respectively. These requirements (bandwidth and delay) are adjusted locally at each node based on the path-length and incoming traffic in static flat wireless network where all the nodes are homogeneous multimedia sensor nodes capable of performing all possible application tasks (video, audio, scalar data) and equipped with single radio interface and multi-channels. QoS-aware packet scheduling policy considers different priorities for real time packets and non-real time packets by using at each node a classifier that checks the type of the incoming packets and sends to appropriate queues, and a scheduler that schedules the packets according to the delay and bandwidth requirements. The proposed protocol -as shown through simulation- clearly improves average delay per real-time packet, average lifetime of a node, and throughput of non-real-time data when compared with single-r and multi-r mechanisms.

A multi-constrained routing algorithm, MCRA, [18] is proposed for multimedia communications over WMSN, which is based on query-flooding and query-driven data delivery mode. The proposed algorithm is designed to provide end-to-end delay and packet loss ratio suitable for multimedia content, and balance the energy consumption. The routing process starts from the sink node that floods the network with interest-messages. The source node that receives those interest messages and matches the needed query, selects the path with minimum hop-count to send its data. Then the sink node when it receives the data packet calculates the coordinates of the source node using the logical coordinates (hop-count). The presented algorithm tries to reduce the amount of flooding (interest messages) by either not forwarding the interests to nodes receives them already or by merging multiple interests in one message.

### 2.2. Security in WMSNs

Several surveys [19–21] have been conducted for security protocols in WSN in general. They discussed the existing security mechanisms and reviewed the proposed security protocols in authentication and encryption, secure routing, key management and distribution, and intrusion detection systems. Most of the Preexisting secure protocols -as shown in this subsection- have very strong requirements, like having different shared keys between each of the nodes and the base station or hardware modification, which are arguably too strong requirements for those proposals to be feasible.The following works discussed security implementations in WMSNs:

A security scheme is proposed in [22] for collaborative image transmission from image sensors to cluster heads where heterogeneous cluster-based wireless image sensor network architecture is assumed. The proposed scheme does not depend on a key-based security management, but it uses an approach for secret image sharing on multiple node-disjoint paths for image delivery. In this approach, the image is separated into overlapped and non-overlapped regions where the later is directly transmitted without encryption after compression, while the former is conveyed via secret sharing and transmitted with appropriate distribution ratio via multiple paths. The proposed scheme also exploits the inter-image correlation among sensors sharing overlapped regions in order to achieve high-security image transmission and to be more energy efficient.

In order to assure authentication and data integrity for multimedia data delivered over WMSN, an energy-aware adaptive wavelet-based watermarking technique for real time image delivery is presented in [23]. This technique embeds additional data called a watermark into some location in an image object so it can be detected later to make an affirmation about the object. The watermarking locations or positions are adaptively chosen by using two thresholds to insert the watermark according to network conditions so that the energy efficiency and security can be achieved. In order to degrade the effect of the distortion of watermarked image, the proposed scheme embeds the watermark into few positions as possible to make it invisible and allocates extra network resources to protect this embedded watermark from high distortion so it can be detectable. In addition, it also embeds watermark coding redundancies into the original image so the watermark becomes more robust to packet loss. The frequency-based (middle band) discrete wavelet transform (DWT) has been selected because it is more robust, easy to recognizable and authenticated at the receiver side, and it reduces computation complexity and process delay by exploiting the correlation of the inter-frames.

In [24], a privacy paradigm called HoLiSTiC is proposed that secures routing and topology information for WMSNs against outsider attacks. The paper assumes a clustered network with some nodes equipped with free-space optical (FSO) capabilities. Also it assumes that the BS and CHs have bidirectional communication links and the camera and transport nodes have unidirectional links. The proposed protocol requires that each node to have an individual key shared with the BS and pairwise keys shared between adjacent visual nodes in a cluster. In addition, every network entity has two pre-deployed network-wide keys. All keys are employed for symmetric cryptography to provide a variety of security services.

A secure data converter architecture is proposed in [25] for WMSN that employs fingerprinting and encryption capabilities for simultaneously digitize and authenticate sensor readings. The proposed architecture suggested hardware modifications to the data converter and aims to reduce the computational complexity of the security algorithms at the aggregation points in the systems that need in-network processing. This can be done by embedding an authenticator payload into the data converter or the modulator output in a way that it is not easily extractable without access to the secret key, and can be used to verify the integrity of the sensor reading.

## 3. Network Architecture and System Model

**Figure 1.**
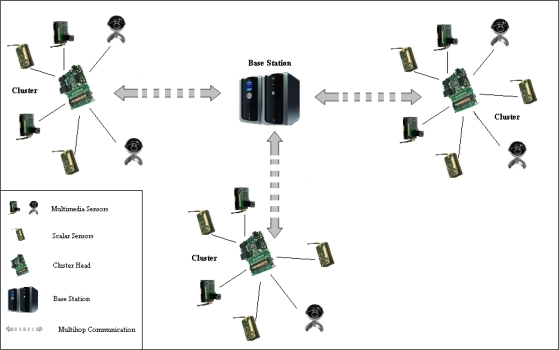
Single-tier Clustered Architecture.

In this section, we briefly describe the network architecture model adopted by our proposed routing protocol, the communication pattern among the nodes, and the assumptions made in implementing both the routing and security algorithms. Our proposed network architecture’s model is following the *single-tier clustered architecture* [1], as shown in Figure 1, deployed with heterogeneous sensor nodes where camera, audio and scalar sensors within each cluster relay data to a cluster head. The cluster head has more resources, more powerful, and it is able to perform intensive data processing. The cluster head is wirelessly connected with the sink or the gateway either directly (in the case of first-level cluster head) or through other cluster heads in multi-hop fashion. The communication among the sensor nodes both within a cluster and the communication between cluster-heads are managed by our proposed cluster-based multi-path routing protocol to efficiently handle multimedia content over the network and maintain the energy consumption of sensor nodes. Nodes within a particular cluster communicate directly with their cluster head in a certain schedule and each cluster head is responsible for performing data aggregation and fusion in order to decrease the amount of transferred data and the number of transmitted messages to the sink. A cluster head is also responsible for selecting a suitable path for each type of data, e.g., paths with good link quality or minimum delay are appropriate for multimedia content, disjoint paths are suitable for multimedia streaming, and paths with less strict QoS conditions can be used for scalar data.

Our proposed routing protocol is based on hop count and received signal strength of the sensory message as an indication on the link quality and distance between the nodes. The level of the received power of a message in a large-scale wireless sensor network can be calculated using the propagation model at physical layer:

(1) $$P_{r}=P_{t}G_{t}G_{r}\;{\left(h_{r}h_{t}\right)}^{2}\;d^{-4}L^{-1}$$

Where *P_t_* and *P_r_* are the power level of transmitted and received message respectively, *G_t_* and *G_r_* are the transmitter and receiver antenna gain respectively, *h_t_* and *h_r_* are antenna height for the transmitter and receiver respectively, *d* is the distance between the transmitter and the receiver, and *L* is system loss factor. Then, received signal strength (RSS) of a message can be equal to:

(2) $$\mathrm{RSS}=P_{r}/P_{t}\to \mathrm{RSS}=G_{t}G_{r}\;{\left(h_{r}h_{t}\right)}^{2}\;d^{-4}L^{-1}$$

If we assume that antenna gain of the transmitter and receiver are equal to 1, antenna height of the transmitter and receiver are equal to 1, and system loss factor also equals 1, then RSS can be approximated as a function of distance between the transmitter and receiver as a dominating factor affecting its value: RSS = 1/*d*^4^. For more accurate propagation model, signal-to-noise ratio (SNR) and bit error rate (BER) should be taken into account [26] along with the received signal strength in order to consider noises (from receiver and environment) and interferences from other packets arrived simultaneously:

(3) $$\mathrm{SNR}=10\;\text{log}\;\left(\frac{P_{r}}{N_{p}+\sum_{i=1}^{n-1}P_{r}}\right)$$

(4) $$\mathrm{BER}=0.5\times \mathrm{er}\;\mathrm{fc}\;\left(\sqrt{\frac{P_{r}\times \mathrm{BW}}{N_{p}\times R}}\right)$$

Where *BW* is the channel bandwidth, *N_p_* is noise power, and *R* is data rate.

With respect to our security scheme, we assume that all nodes have unique IDs and the base station is trusted node during all network operation time and thus it can keep the security keys in its memory. Also we assume that the time required for establishing secure links (at the route discovery phase) is smaller than the time needed by an adversary to compromise a node during node deployment.

## 4. Cluster-Based Multipath Routing in WMSN

This section describes the routing operation of our proposed cluster-based multi-path routing protocol for WMSNs, called SCMR, which is based on the hierarchical structure of multiple paths established depending on hop count and received signal strength (along with measured SNR & BER) as an indication on the link quality and distance between the nodes. SCMR depends on the local information exchanged among the nodes to establish the routes to the sink and does not require any coordination measurement equipments or position message exchange.

### 4.1. Route Discovery

We focus here in demonstrating the mechanism of establishing our proposed routing protocol, and in the next section we explain the process of securing the routing protocol. We are using two performance metrics: hop-count (as indication for distance from the sink and delay), and received signal strength (combined with SNR & BER) as indication for link quality (interference and noise level) and distance from the sender. Two thresholds (upper, and lower) are used in SCMR to compare the signal strength value of the received packets, where the upper threshold is used to determine the 1st-level cluster head and group member nodes (as described below) and the lower threshold is used to establish the links between cluster heads.

**Figure 2.**
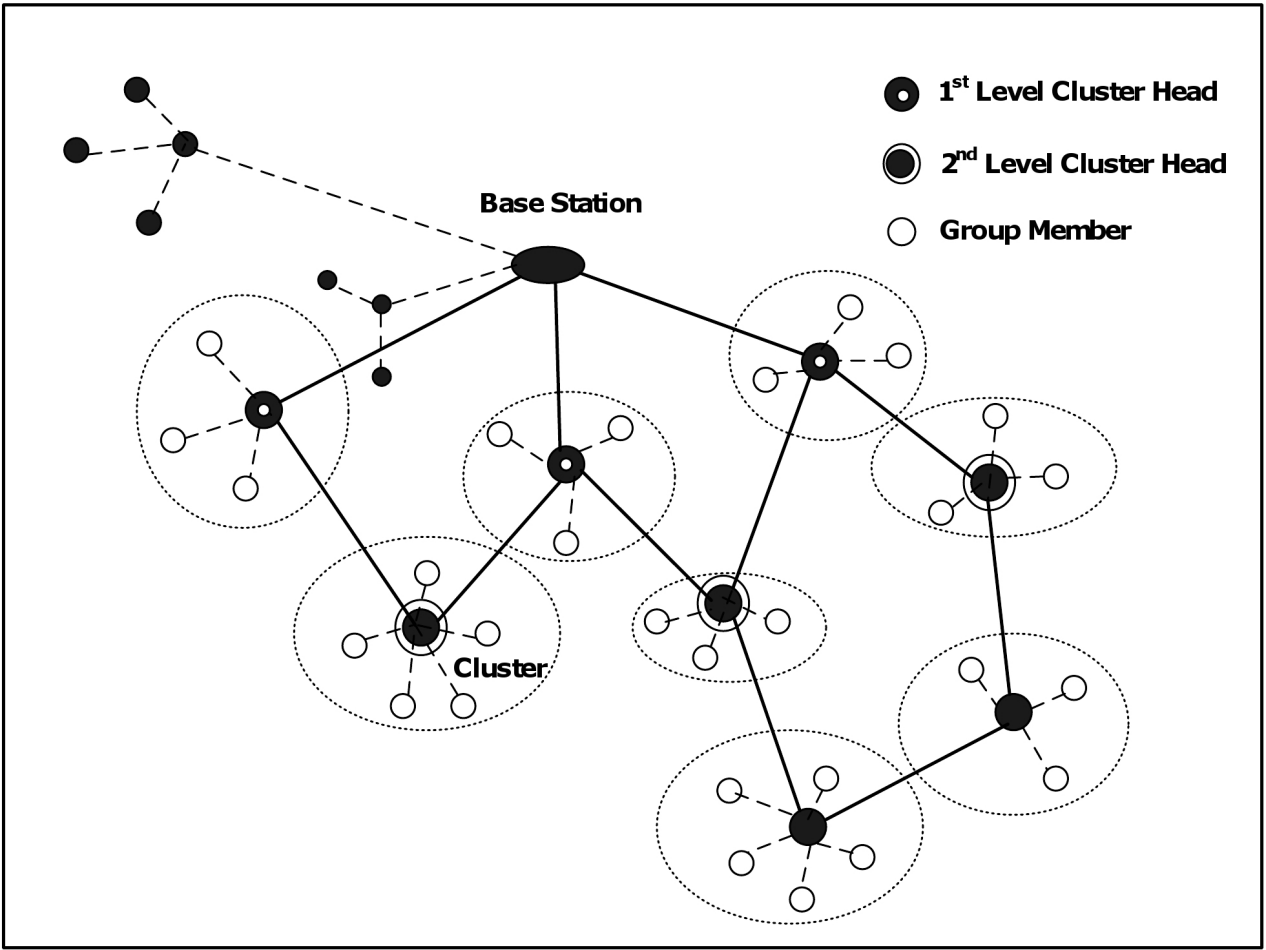
A Simple Example of Cluster-Based Multipath Network.

In the initializing phase of our proposed routing protocol, the base station starts sending periodic broadcast messages, called BS-Msg, to the surrounding nodes. BS-Msg contains the identification number (ID) of the base station and the relevant security information to authenticate the communication with other nodes. The nodes that receive BS-Msg messages compare the received signal strength index (RSSI) with the upper threshold (Thr-High). If RSSI is greater than Thr-High, these nodes respond to the base station by sending back acknowledgment messages informing their joining the base station as their parent. Then, they start acting as 1st-level cluster heads as shown in an example in Figure 2 and broadcast periodically control messages called CH-Msg to their neighboring nodes. CH-Msg contains the ID of the cluster head, number of hops between the cluster head and the base station in the current found path, IDs of the nodes joining this path up to the current cluster head, and the relevant security information. For each CH-Msg received by the surrounding nodes of the 1st-level cluster-heads, RSSI is measured and compared with two thresholds, Thr-High and Thr-Low.

If the signal strength of the received CH-Msg is greater than Thr-High, the receiving node will start behaving as a group member and sending back an acknowledgment message informing its joining to the corresponding cluster head. Receiving a CH-Msg with RSSI greater than Thr-High indicates that the sender (CH) is in near region, as seen from Equation 2, and the quality of the link is good and thus this CH can better serve the communication toward the base station. In case a node receives more than one message from different CHs with received signal strength larger than Thr-High, the node selects the cluster head of the highest signal strength value.

**Figure 3.**
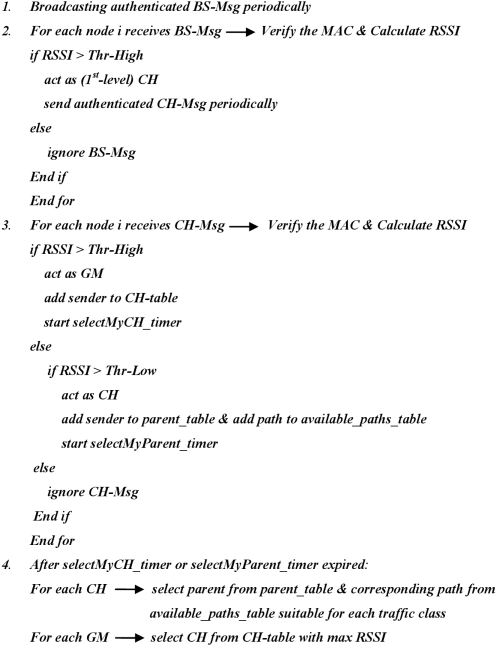
Pseudo-code of the Main Part of the Routing Protocol.

The nodes that only receive messages with received signal strength between Thr-High and Thr-Low will start acting as new cluster heads, in this case 2nd-level cluster heads, and respond back to the sender informing their selection of him as one of their possible parents toward the base station. New cluster heads may receive different CH-Msgs from previous-level cluster heads. In this case new cluster heads consider these messages in order to construct multiple paths toward base station and sort these paths based on certain criteria (such as link quality, end-to-end delay, bandwidth, or number of hops in the path). Paths with good conditions, like high link quality, short end-to-end delay, enough bandwidth, or less number of hops, are reserved for multimedia communication that requires certain level of quality of service requirements. Other paths will be used for other types of data that does not require strict QoS requirements such as scalar data. If the signal strength is less than Thr-Low, the message is considered as lost or ignored. This process continues in the same manner to build the network until all nodes join the network and determine their rules, *i.e.*, cluster head or group member, and all possible paths are found. The main routing process can be outlined in the following pseudo-code (as shown in Figure 3).

### 4.2. Route Optimization and Local Repair

**Figure 4.**
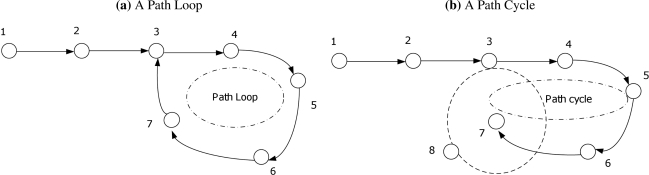
Examples of Path loop and Path Cycle.

In order to optimize the found routes in route discovery phase, path loops and path cycles should be prevented. For *path loops*, each CH that receives CH-Msg from other nodes checks first the IDs of the nodes joining the path to know whether it already joined this path before or not as shown in Figure 4(a). If a CH receives CH-Msg belongs to one of the paths already found before, it checks the conditions and the status of the given path in order to update its routing information about this path and reflects these changes (if any) on its decision of selecting the proper path for each type of data. Moreover, for path optimization with minimum number of hops, a CH checks for every given path whether it is a child for any participating node in this path (except of course its direct parent in this path). In this case, it is better for the CH to communicate directly to that parent instead of making a path cycle as shown in an example in Figure 4(b). Thus, if a *path cycle* is found, the cluster head deletes this path from its routing information (as it is just a longer version of already found path) and keeps the shorter path.

In order to detect any node or link failure, an acknowledgment system has been used in the network. Each source node or intermediate CH keeps track of sent data packets toward the sink. After receiving a certain number of data packets, a CH sends an acknowledgment message (Ack-Msg) to the sender and in the same manner waits an Ack-Msg from its parent confirming receiving the data packets. If a node did not receive an Ack-Msg from its parent (or its CH), it will assume that there is a node-failure or link-failure and it will select another parent (path) -depending on its routing information tables - suitable with the current type of data. Notice that in this process there is no need to initialize the entire network to overcome the existing failure; it just affects the nodes along the failed path and because of that it is called *local repair*. Then the source node starts sending its data again to the base station through the new parent. Notice that in this case, the affected CH will reflect the changes on its next CH-Msgs in order for all participating nodes in the old path to update their routing information according to the new state of that path.

### 4.3. Two-Level QoS-Aware Scheduling

**Figure 5.**
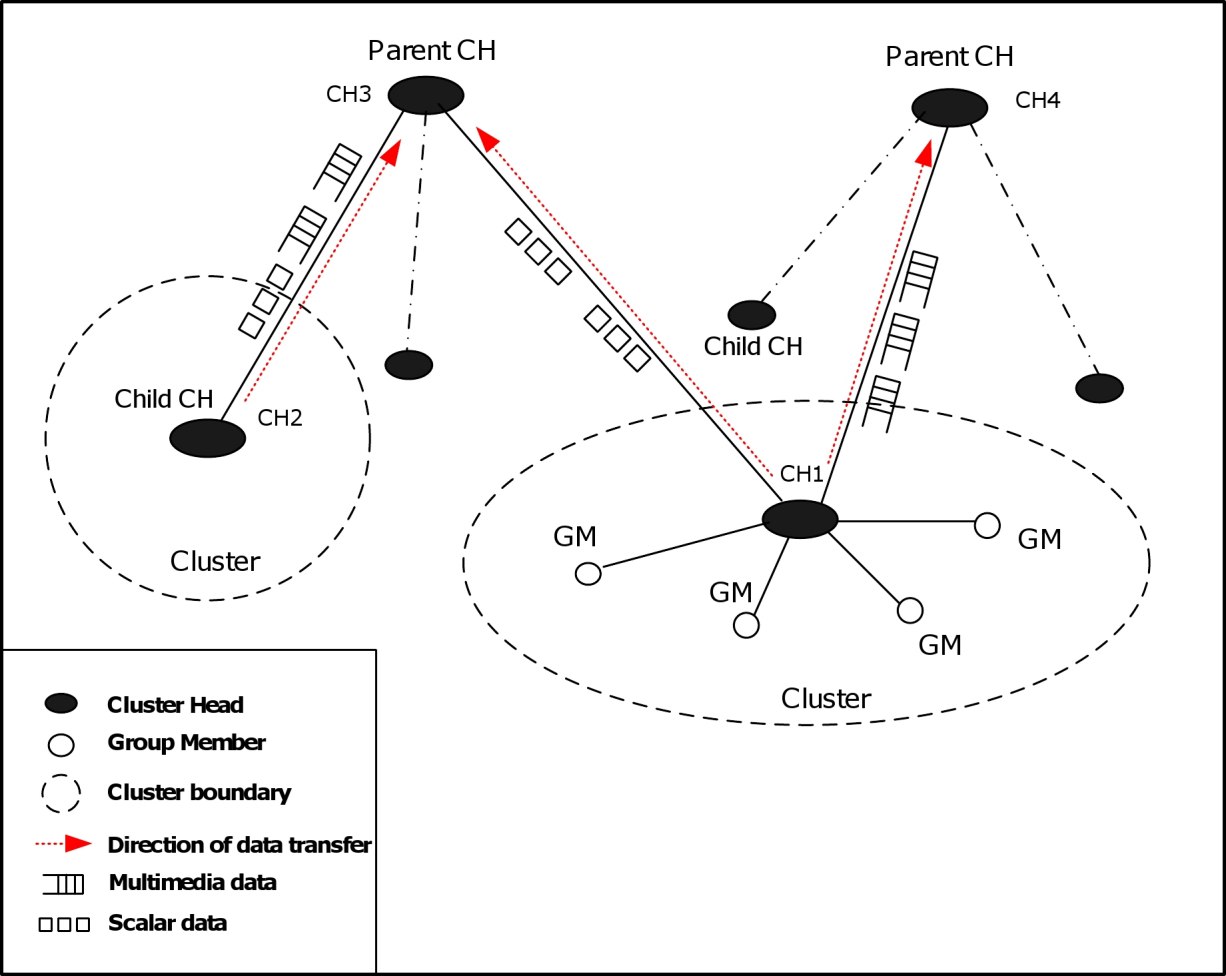
A Simple Example of Two-level Scheduling.

After establishing the network, all group members (sensor nodes) in each cluster are assigned to a cluster head and each cluster head in the network knows now its parents toward the BS (for multiple paths). Before data transmission, we introduce two-level QoS-aware scheduling: low-level scheduling within the cluster among the group members, and scheduling among the cluster heads at higher level in order to increase the packet delivery ratio and throughput for multimedia data. The two-level scheduling is shown in a simple example in Figure 5. We prefer to adopt TDMA protocol to access the channel as it has a natural advantage of collision-free medium access, and it is more appropriate for transmitting multimedia applications with QoS at reliable channel conditions and heavier traffic load. In order to avoid channel under-utilization and to decrease the delay, dynamic time-slot is assigned to the nodes depending on the amount of data to be transmitted and the time for sending Ack-Msgs if needed.

At low level, before GMs start sending their different types of data to their CHs, each CH should schedule the data transmissions among its GMs within the cluster in order to give higher priority to the nodes that demand higher or strict QoS requirements for their data, and to avoid collisions and interferences at receiver side. The low-level scheduling process is initiated by the CH by sending a broadcast message asking each GM in the cluster to send a request message (Req-Msg) informing about the type of data to be transmitted, its amount, and its requirements (such as playout deadline, BW... *etc.*). This broadcast message, Assign-Msg, contains the control slot assignment, based on TDMA (*i.e.*, time slot to each node), to each GM in the cluster to send its Req-Msg. The duration of the time slot is enough to any node in the cluster to send its Req-Msg and the time slot is unique for each node to avoid collisions. During the request phase, each GM sends a Req-Msg to its CH at the allocated time slot informing about the data to be sent (if available) and its QoS requirements.

**Figure 6.**
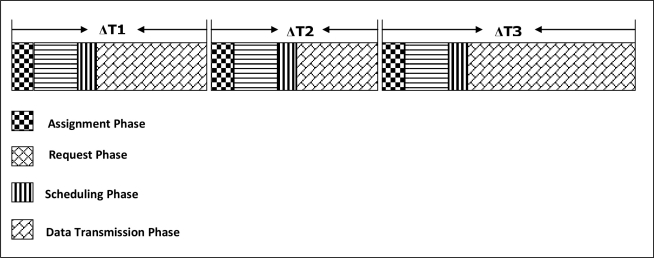
A Cluster Time Intervals for Scheduling Process.

Then based on the collected information from the request phase, each CH generates a transmission schedule for the active GMs and distributes it in the cluster. The resulting schedule is sent to all GMs by broadcasting a scheduling message, called Sched-Msg, to inform each GM with the specified time schedule for sending each type of data. The duration of the time-slot depends on the amount and type of data to be transmitted as requested by each GM. By this way, multimedia streaming and time-critical data can be transmitted first, then less priority data such as still images and then scalar data can be sent later. Moreover, for better energy efficiency, GMs can turn off their radio transceiver when the schedule has been received until the time slot for transmission a certain data type approaches or to the end of the data transmission phase if they are passive nodes. After receiving the schedule, each GM will transmit its data during the assigned time slots for each data type and the CH sends, after receiving a certain number of data packets, an Ack-Msg to the sender as described before. When the data transmission phase complete, a CH sends again the Assign-Msg to its GMs to send their requests. A cluster time-intervals is in Figure 6 illustrating the scheduling operation.

At higher level, each intermediate cluster head—in the same manner done at low level—schedules the traffic toward the sink from other cluster heads (its children) based on the type of the data and its QoS requirements. For example, as shown in Figure 5, CH1 selects the path through CH4 to send its streaming multimedia data and the path through CH3 to send the other types of data based on the proposed routing algorithm, while CH2 sends all its data to CH3. CH3 and CH4 then need to schedule the transmission from children CHs following the same steps done at low level inside the cluster.

## 5. Light-Weight Distributed Security Scheme

In this section, we explain in details the implementation of our proposed lightweight distributed security scheme of key management: Our security protocol provides privacy and integrity against external attacks. It also limits the effect of insider attacks *i.e.*, nodes that have been compromised and controlled by an attacker who now possesses all valid keys) since the generated keys are unique and cannot be used in different areas of the network. In addition, our security scheme allows for aggregation processing since cluster heads can decrypt the sent data if necessary and update the corresponding information. This is achieved with the shared keys between the nodes in the cluster. Moreover, our proposed security algorithm supports both authenticated encryption and authentication only services: with authenticated encryption, the data payload is encrypted using an encryption algorithm such as MISTY1 or Skipjack algorithm [27] and the entire packet is authenticated with a message authentication code (MAC) using for example Hash-based Message Authentication Code (HMAC) based on cryptographic hash function or message digest (SHA1 [28]). The MAC is computed over the encrypted data and the packet header. The decryption operation is done only at the sink and at cluster heads during aggregation process (if needed). In authentication only service where the data is not sensitive, our security scheme authenticates the entire packet with a MAC tag, but the data payload is not encrypted. The notations shown in Table 1 are used for establishing formulas of the needed security keys, as described in the following subsections:

### 5.1. Key Management

Our security scheme uses symmetric keys to encrypt and authenticate messages. Therefore, sensor nodes will only receive messages from other nodes that share the same security keys. The key management is composed of two phases: a key generation phase and a key distribution phase. In the *key generation* phase, a Master key (K*_m_*) is installed in the sensor motes when they are programmed with the intended software. The *key distribution* phase provides a means for distributing or synchronization of the security keys (unique-node key, pair-wise key, and unique-cluster key) among the nodes. The key management protocol satisfies the following security properties of a key establishment:

- The shared security keys between a node and the neighboring nodes and/or BS cannot be used to recover the master key. This is guaranteed because of the one-wayness of a secure hash function.
- The master key (K*_m_*) is kept by the nodes as long as it is necessary to establish security keys with their neighbors, and then it is deleted from their memory. The base station holds K*_m_* along with the unique-node keys of each sensor node in the network.

(1) The **unique-node key** (K*_i_*) is the shared key between every node (i) in the network and the trusted base station and used to verify node identities and exchange data securely. The derivation of this key for each node is as follows:

(5) $$K_{i}=F(K_{m},S_{i})$$

where F( ) is a secure hash function, K*_m_* is the master key and S*_i_* is the unique identifier (number) for the i-th sensor node.

(2) The **pair-wise key** (K*_ij_*) is the shared key between two neighboring nodes i and j, such as each GM and its CH in a cluster and every CH and its parent in the network, and it is computed as follows:

(6) $$K_{\mathrm{ij}}=F(K_{m},S_{i},S_{j})\;\;\;\;\;\;\;\;\;\;\;\mathrm{where}\;S_{i}<S_{j}$$

Notice that since both nodes have the master key, they both can compute the pair-wise key. This key now can be used to send information from S*_i_* to S*_j_* (and vice versa) in a secure and authenticated manner. Also this key is needed especially for aggregation process and data fusion at the CH. In order to support aggregation process, CHs need to be able to look at data sent by their GMs and perform some function on the data if necessary.

(3) The **unique-cluster key** (K*_ch_*) is the shared key within a cluster in the network and it is different from other cluster-keys of other clusters. This key is used for command dissemination and broadcasting messages and it is generated as follows:

(7) $$K_{\mathrm{ch}}=F(K_{m},S_{i},S_{i})$$

where S*_i_* is the unique identifier number for the cluster head. Once the unique-cluster key is settled, node i can broadcast commands/messages to all the nodes in the cluster encrypted and authenticated using K*_ch_*.

### 5.2. Implementation of the Security Scheme

In this subsection, we will focus on the security scheme and authenticating the participating nodes while establishing the paths in the clustered WMSN network. As we mentioned before, all the nodes in the network have the preloaded master key (K*_m_*), then each one of them compute the unique-node key shared between the node and BS. Before BS starts broadcasting its BS-Msgs, it first authenticates itself by properly calculating the MAC tag of these messages using the master key (K*_m_*) and including this MAC in one of the fields of BS-Msg, as follows:

(8) $$\mathrm{BS}\to S_{j}:\mathrm{BS}-\mathrm{Msg},\;{\mathrm{MAC}}_{\mathrm{Km}}\;(\mathrm{BS}-\mathrm{Msg})$$

Upon receiving this message, the node S*_j_* checks the MAC using the same master key before processing the other data to extract the routing information. If the MAC is verified, BS will be added in the routing table of node S*_j_* along with all relevant security information such as nonce identifier (random number used once composed of random number and time stamp) and sequence number used to avoid replay attacks, and pair-wise key will be generated. On the other hand, when the verification process is valid, these nodes reply to BS by sending back Ack-Msgs authenticated with MAC using the unique-node key to avoid impersonation attempts and to authenticate themselves to the BS as 1st-level cluster heads. In the same manner, cluster heads include in their CH-Msgs the MAC tag using the master key and other relevant security information in order to authenticate the communication between them and other cluster heads and their group members and to exchange data securely:

(9) $$\mathrm{CH}\to \mathrm{BS}:\mathrm{Ack}-\mathrm{Msg},\;\mathrm{MA}C_{K_{i}}(\mathrm{Ack}-\mathrm{Msg})$$

(10) $$\mathrm{CH}\to S_{j}:\mathrm{CH}-\mathrm{Msg},\;\mathrm{MA}C_{K_{m}}(\mathrm{CH}-\mathrm{Msg})$$

At the same way, each node S*_j_* behaves as a group member in any cluster of the network sends back an Ack-Msg to its CH which is also properly authenticated by including the MAC tag using the pair-wise key.

(11) $$S_{j}\to \mathrm{CH}:\mathrm{Ack}-\mathrm{Msg},\;\mathrm{MA}C_{K_{\mathrm{ij}}}(\mathrm{Ack}-\mathrm{Msg})$$

After the network is built up and each node knows its parent (if it is a cluster head) and its cluster head (if it is a group member), all the cluster heads and the group members compute the unique-cluster key of their clusters. This key will be used later on for authenticating any cluster head messages for broadcasting messages or disseminating commands within the cluster. Finally, the master key should be deleted from the memory of all nodes since the security of our protocol depends on the deletion of the master key at the end of the process. We should take care that the deletion is unrecoverable, for example by overwriting the master key (in practice several times). Now, the communication between the nodes and the base station has been secured and the sources can send their data securely:

(12) $$S_{j}\to \mathrm{CH}:\mathrm{Data}-\mathrm{Msg},\;\mathrm{MA}C_{K_{\mathrm{ij}}}(\mathrm{Header}|E_{K_{\mathrm{ij}}}(\mathrm{Data}))$$

### 5.3. Node Addition

Notice that after deletion of the master key from the network, we will encounter a problem for node addition, node movement, or when a path condition changes during a local repair or rediscovery process. In all these cases, we cannot establish a new connection between two nodes that they did not communicate before without having the appropriate shared security keys between them. And in order to generate these keys, the nodes should know the master key that is already deleted from their memory.

One solution to this problem is to assume that the base station is secure and trusted at any time during the network operation, so that there is no need to delete the master key from its memory. In the case of node addition, a new node has the master key but the existing nodes do not have. So, the new node first generates the unique-node key from the master key and waits until it hears broadcast messages (CH-Msg) from other CHs, then it selects one of them, or more depending on the proposed routing protocol, as its CH or parent toward BS. The new node then sends a request message authenticated with the MAC tag using the unique-node key to the selected CH asking the base station for the appropriate keys (pair-wise and unique-cluster keys) for the new connections. The selected CH forwards the request message as it is (without verifying the MAC) toward BS. Then, BS verifies the MAC of this message, generates the requested security keys using the master key, and sends them back to the requesting node and its CH or parent. The new node then establishes the new connections and deletes the master key from its memory.

For node movement or when the path conditions have changed, the same process of adding new nodes will be followed except that these nodes do not have the master key as well but they have already generated the unique-node keys.

## 6. Performance Evaluation

In order to evaluate the performance of the secure cluster-based routing protocol, several simulation experiments (over 100) with various random topologies were run. We implemented our proposed protocol using NS-2 version 2.34. NS-2 [29] is an open source, discrete event simulator which is widely used for research purposes. It has an excellent implementation of the 802.11 standards at physical, Data link and higher layers. We simulate the proposed routing protocol assuming a multi-hop network of size 500 m × 500 m deployed with different number of sensor nodes ranging from 50 to 200 in randomized grid. The sink is located in the center of the network. The traffic is CBR of 600 packets/sec and the packet size is 316 bytes. Table 2 shows our simulation environment and other parameters used in our simulation.

### 6.1. Routing Performance Analysis

**Figure 7.**
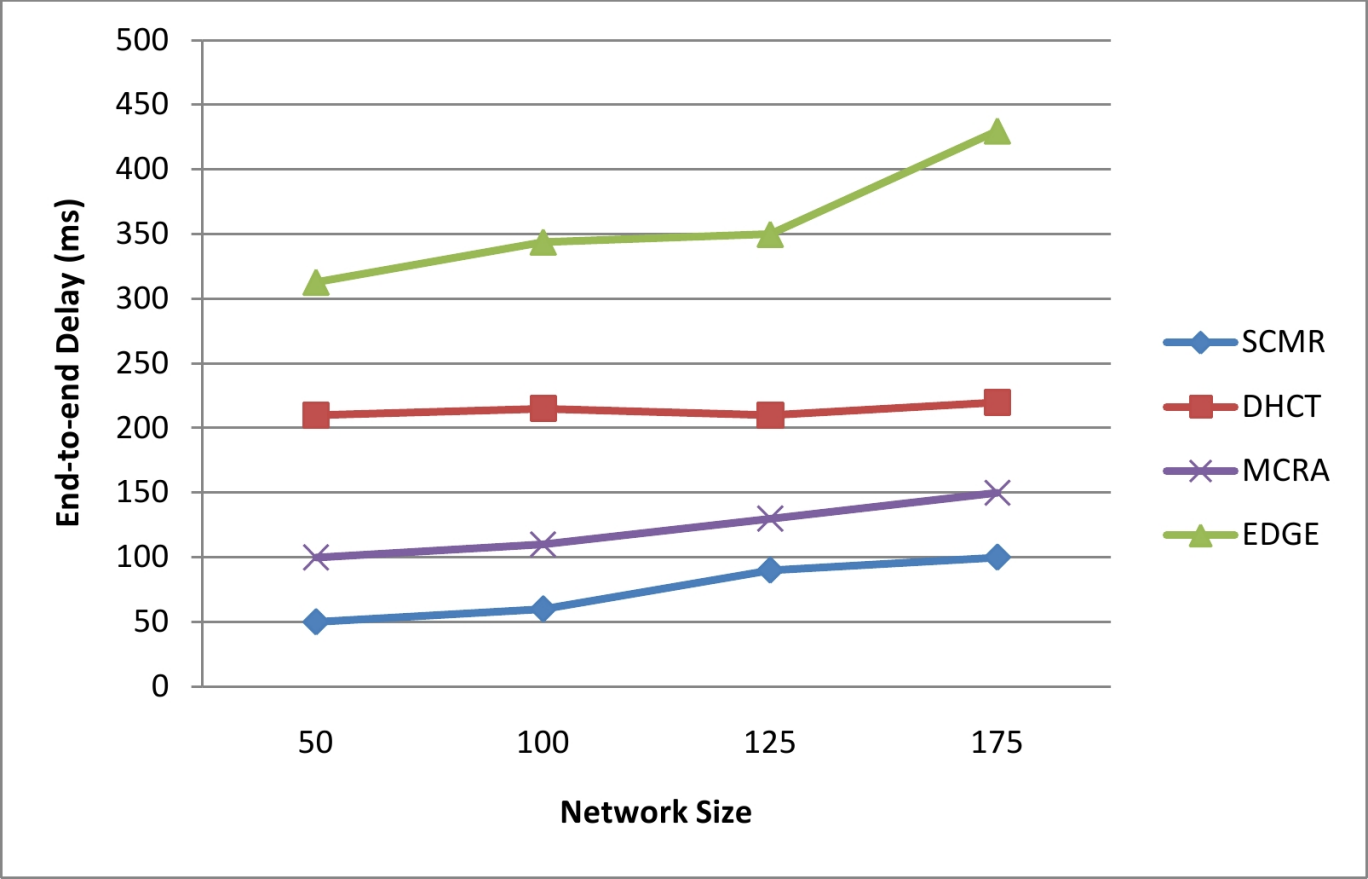
End-to-End Delay of Our Protocol Compared with Other Protocols.

In this subsection, we compare our performance evaluation results (average) with DHCT [15], MCRA [18], and EDGE [16] protocols. We consider in this comparison four important performance metrics: throughput, end-to-end delay, packet delivery ratio, and power consumption:

End-to-end delay is one of the important QoS parameter that we consider in designing our proposed routing protocol to handle the real-time traffic and deliver the packets within their playout deadlines. End-to-end delay is the time difference from the time a source node sends its data packet to the time the sink receives it, and it can be measured as sum of (transmission delay, propagation delay, queuing delay, and processing delay at each hop). We obtain an average end-to-end delay of 75 ms which satisfies the end-to-end delay requirements of real-time multimedia packets. Figure 7 shows a comparison between our proposed protocol, SCMR, with the other protocols (DHCT, MCRA, and EDGE) in terms of average end-to-end delay with different node number. It can be seen clearly that our protocol outperform other protocols due to the hierarchical architecture of powerful cluster heads that always select the route with lowest number of hops of better link quality and hence minimum delay.

**Figure 8.**
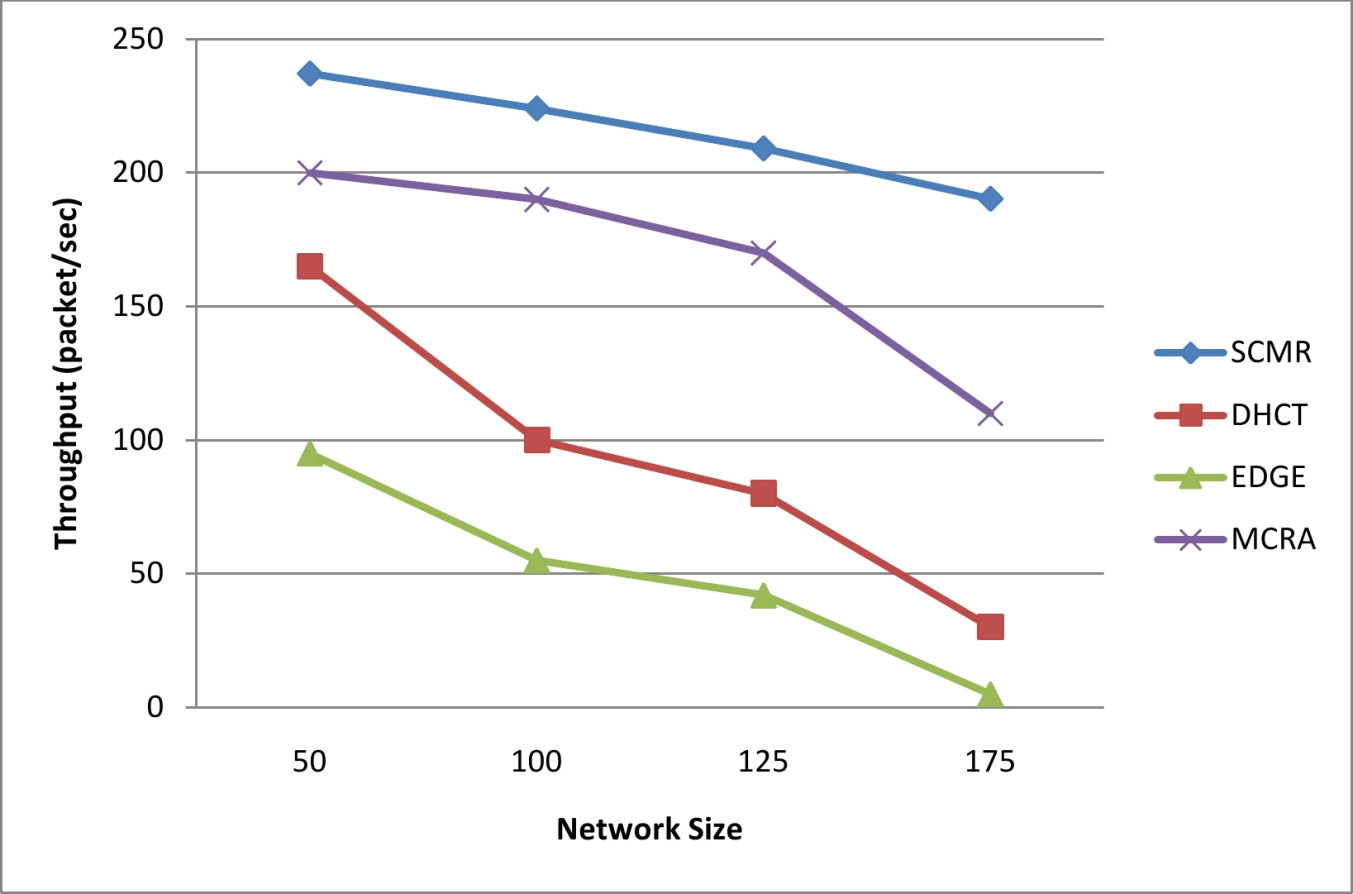
Throughput of Our Protocol Compared with Other Protocols.

In Figure 8, the mean throughput of our protocol is shown compared with the other protocols. The throughput is measured as the total number of packet received at the sink over the simulation period. Selecting multiple paths of better link quality and minimum delay leads to load balancing and efficient utilization of the wireless spectrum, and hence achieves higher throughput and much better performance than other protocols as seen in Figure 8.

**Figure 9.**
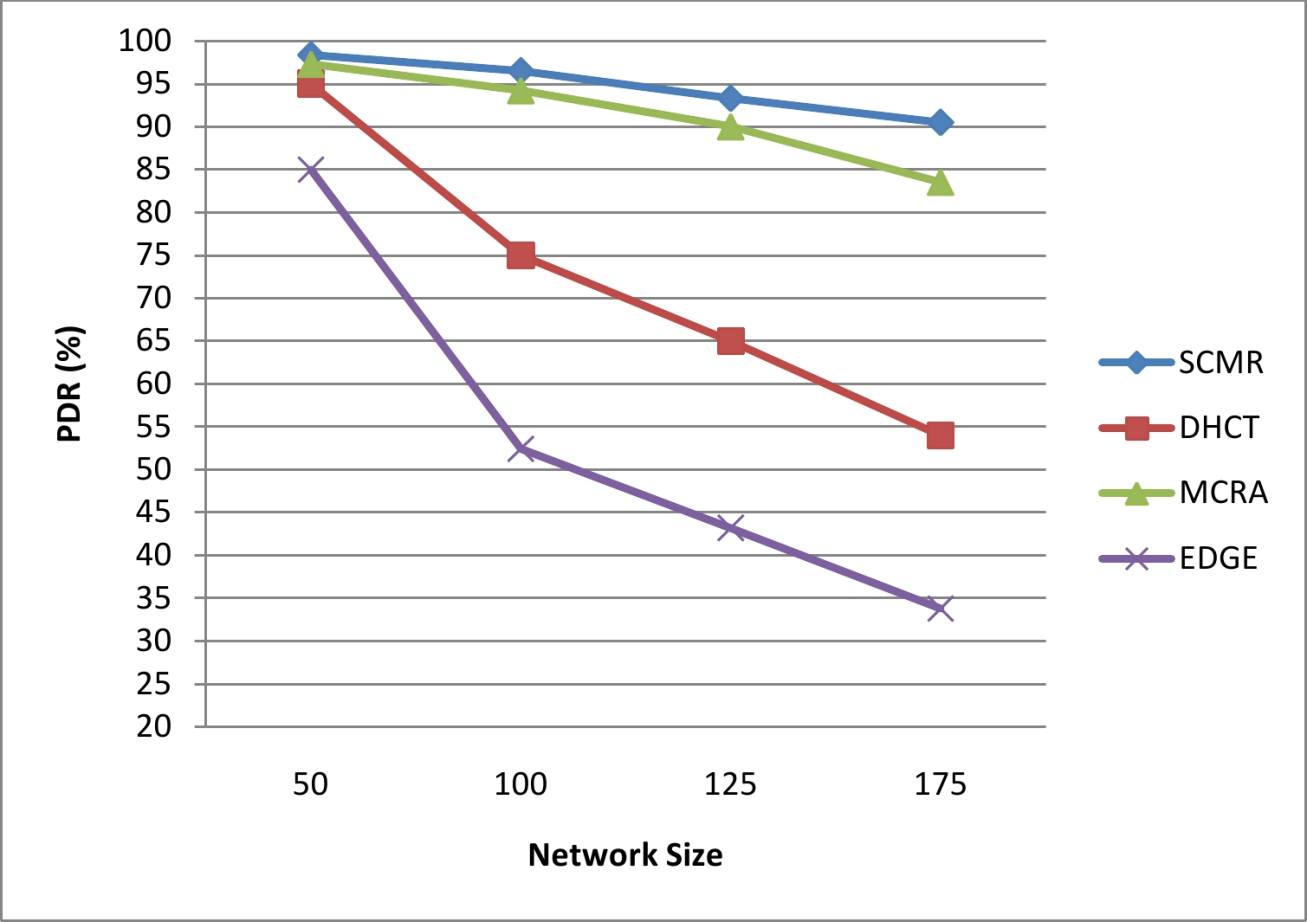
PDR of Our Protocol Compared with Other Protocols.

Figure 9 shows the average packet delivery ratio (PDR) of our protocol with different node number. PDR is measured as the total number of data packets received at the sink over the total number of data packets sent by all sources in the network. It is shown that our proposed protocol outperforms the other protocols, which confirms the previous result, due to the use of the 2nd-level scheduling that prevents collisions and minimizes interference along with the selection of paths with better link quality based on the received signal strength (along with SNR and BER). Also, the use of the fast mechanism of local repair through the acknowledgment system minimizes the effects of any node failure or link break and hence decreases the number of lost packets.

**Figure 10.**
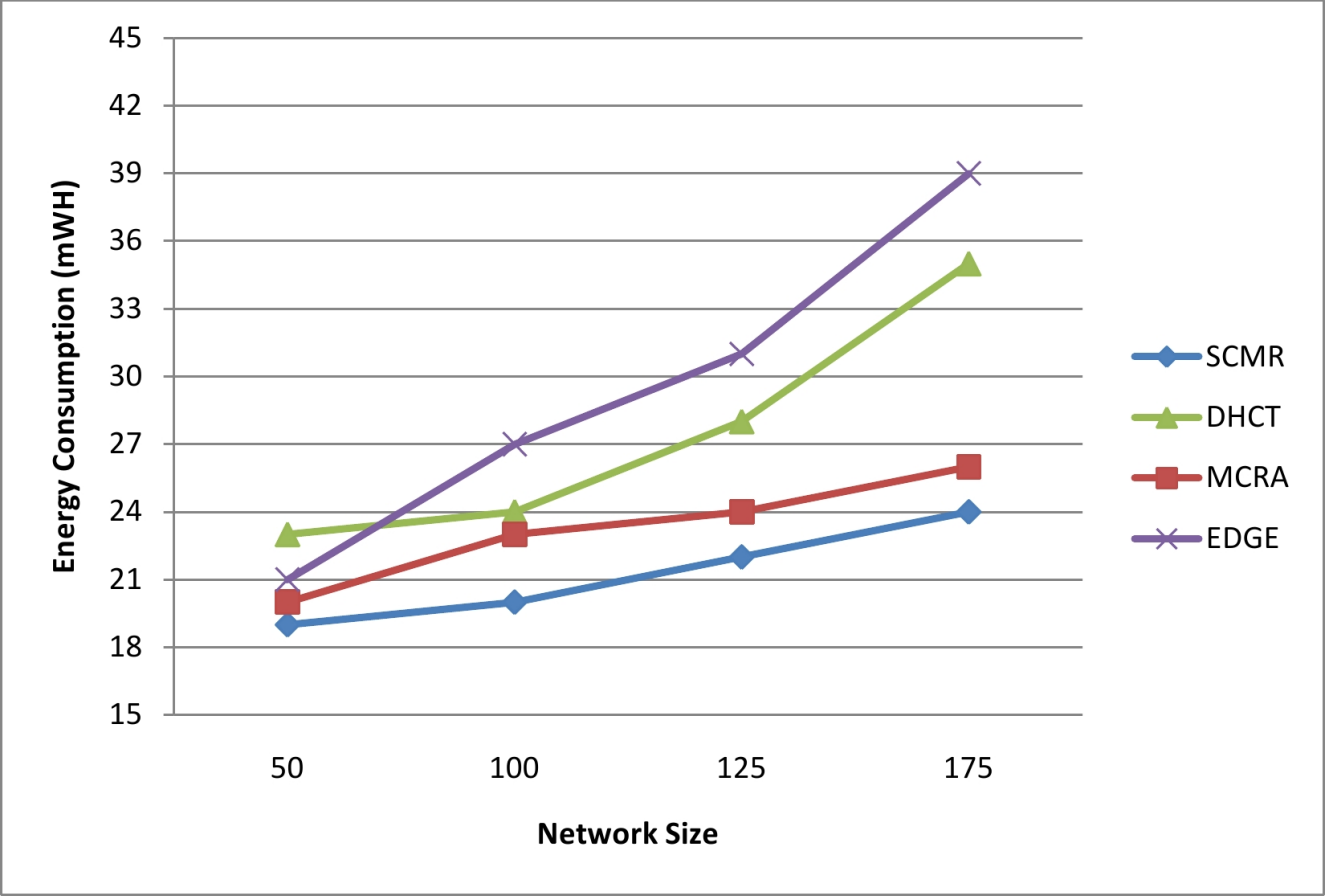
Energy Consumption of Our Protocol Compared with Other Protocols.

Average energy consumption is shown in Figure 10 where we can realize that our proposed protocol SCMR has less energy dissipation comparing with other protocols (DHCT, MCRA, and EDGE) with different node numbers. This result due to the fact that most of the nodes in the clustered network are GMs and need only to communicate with their CHs regardless of the number of nodes in the cluster. In addition, the 2nd-level scheduling prevents packet collisions and interferences and hence there is no need for retransmission lost packets. Also, the paths found by SCMR are optimized in terms of number of hops since the routing algorithm depends on the hop-count as one of its metrics along with preventing path loops and path cycles, which lead to minimum number of packet forwarding from a source to the sink. Moreover, the aggregation process and data fusion done at CHs reduce the size of correlated data within a cluster and thus decrease the needed amount of energy to deliver them.

### 6.2. Security Performance Analysis

In this subsection we examine the security protection achieved by our proposed security scheme against some general security threats in sensor networks, and its performance in terms of memory requirement and scalability:

**Defenses from outsider attacks:** Most of the outsider attacks against WMSN routing protocols can be prevented by providing confidentiality (through encryption and authentication) using the shared security keys. Even a simple scheme uses only the shared master key will prevent unauthorized nodes from joining the topology of the network and hence attacks like selective forwarding, acknowledgment spoofing, wormhole, and sinkhole attacks are disallowed. Hello-flood attacks are detected since broadcasting messages in the network done only by using the master key and unique-cluster key. Also by using the unique-node and pair-wise keys, attacks such as Sybil attack is prevented because a single node cannot present multiple identities without having the security keys. The network is also protected against replay attacks as all messages exchanged in the network are tracked by a time stamp and sequence number. Notice that a GM needs only to maintain a one counter for its CH, and a CH needs to keep counters only for its GMs and parents.

**Defenses from insider attacks:** Security mechanisms using only the master key cannot protect the network against insider attacks or compromised nodes. Insider attacks can disrupt the network by spoofing or altering routing information, selective forwarding, and broadcasting Hello-floods. Therefore, our proposed security scheme can protect the network against insider attacks by verifying both node identities and bidirectional link, as well as authenticating broadcast messages. Identities can be verified by sharing a symmetric unique-node key for every node with a trusted base station. Two neighboring nodes can authenticate the bidirectional link between them by using the master key to verify other identity and establish a shared pair-wise key for securing the communication between them. Broadcasting can be authenticated by using a unique-cluster key derived from the master key and shared, for example, within a cluster or group of nodes. Using those unique symmetric keys, our security scheme resists against insider attacks and minimizes their effects as a compromised node disturbs only a local part and the rest of the network remains secured.

**Figure 11.**
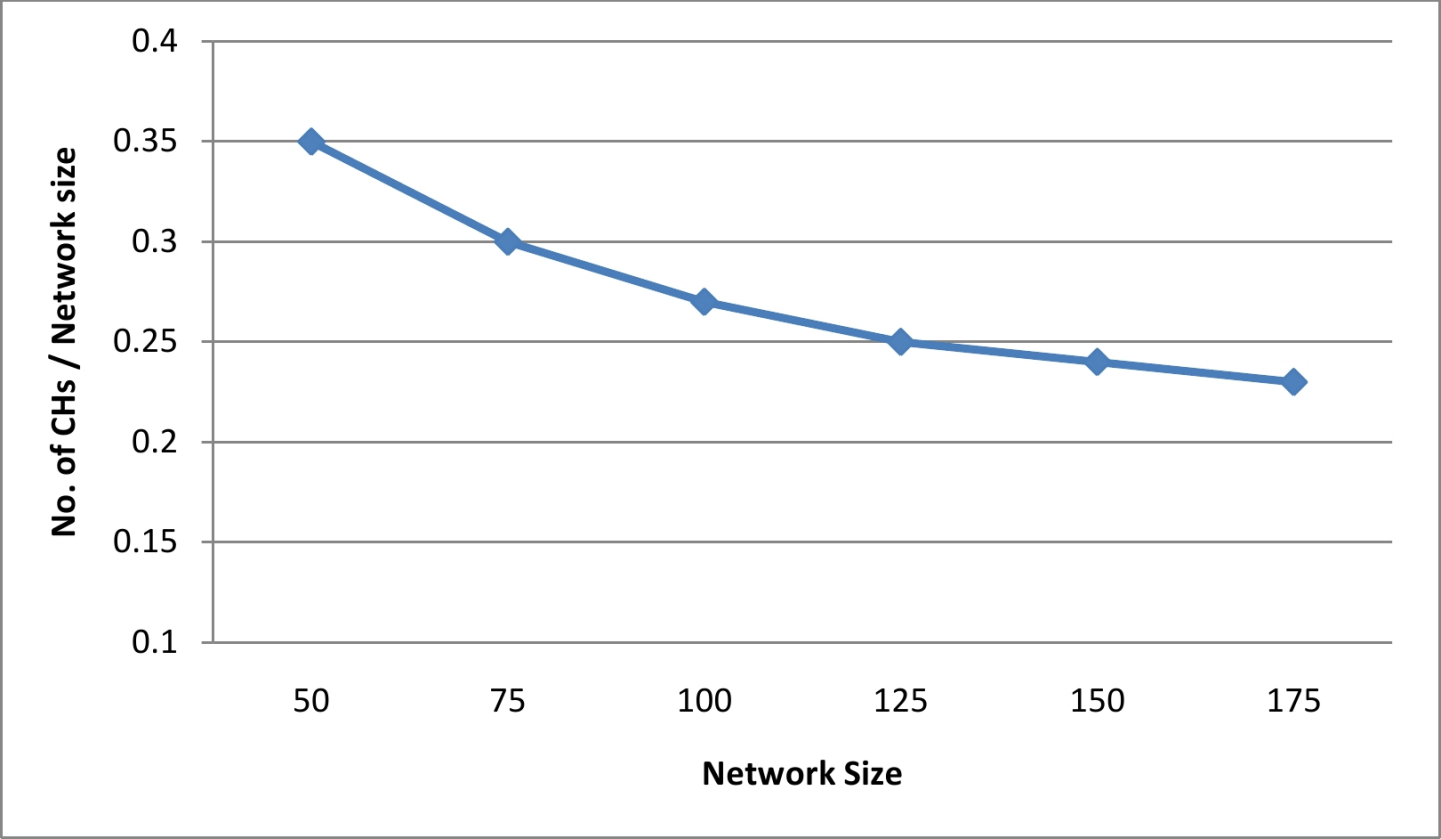
Density of Cluster Heads vs Network Size.

**Scalability:** Our security scheme scales well as it requires only local information for key management without needing of central distribution. Furthermore, the number of security keys needed to be stored at each node does not depend on the network size but only on node density (*i.e.*, average number of group members within a cluster and number of neighboring cluster heads). Average number of GMs or the size of cluster can be determined by adjusting the value of Thr-High. Also it is shown in Figure 11 that the density of cluster heads in the network is decreasing with the network size and tends to be fixed at large network size.

**Memory requirements:** The majority of nodes (*i.e.*, GMs) need only to store three keys: unique-node, pair-wise, and unique-cluster keys. On the other hand, each CH needs to store the pair-wise keys it shares with its group members and parents, in addition to the unique-node and unique-cluster keys. Recall the powerful capabilities of CHs, storing N keys (*N* = *K_i_* + *K_ch_* + (*n* + *m*) × *K_ij_* where n is number of parents and m is cluster size), with an average number of parents is 6 (*i.e.*, six different paths) and an average cluster size of 10, does not need considerable memory space.

## 7. Conclusions

In this paper, we proposed a Secure Cluster-based Multipath Routing protocol (SCMR) for WMSNs designed to handle the additional requirements of reliable data delivering of different traffic classes and provide load balancing by using multipath routing. The proposed routing protocol, SCMR, is based on the hierarchical structure of multiple paths established depending on the hop count and received signal strength as an indication on the link quality, delay, and distance between the nodes. SCMR maintains minimum end-to-end delay suitable for real-time and non-real-time data packets to meet their playout deadline, and achieves high throughput and packet delivery ratio by selecting the paths with better link quality and avoiding collisions and interferences. SCMR reduces energy consumption at sensor nodes by moving the multimedia processing complexity as well as the aggregation process to the cluster heads’ side along with preventing path loops and path cycles in establishing the routes. Also we presented a light-weight distributed security implementation of key management for supporting a secure communication among the nodes. Performance evaluation results show that SCMR clearly outperforms the preexisting ones (DHCT, MCRA, EDGE) in all average end-to-end delay, throughput, packet delivery ratio and battery power consumption. In future work, we will consider a cross layer design optimization especially between the routing and MAC layers to maximize the overall network performance with minimum energy consumption, reliable delivery, and efficient resource management. Also with respect to the security scheme, we will develop a light-weight intrusion detection system to eliminate the threats from insider attacks.

## Figures and Tables

**Table 1.** Security Notations used in Security Formulas.

**Notation**	**Meaning**
**K_ij_**	Symmetric key shared between node i and j.
**MAC_K_(M)**	Message authentication code of M using key K.
**E_K_(M)**	Encryption of M using key K.
**S_i_**	Identification number of node i.
**M_1_|M_2_**	Concatenation of M_1_ and M_2_

**Table 2.** Simulation Parameters.

**Parameter**	**Value**
**Simulation time**	1000s
**Network size**	500x500m^2^
**Node number**	50 – 200
**Link layer**	LL
**Mac layer**	IEEE802.11
**IFQ type**	Queue/DropTail/PriQueue
**IFQ length**	10
**Antenna type**	Antenna/OmniAntenna
**Physical type**	Phy/WirelessPhy
**Channel type**	Channel/WirelessChannel
**Energy model**	EnergyModel
**Bandwidth**	2MB
